# The Effect of Electrophoretic Deposition Parameters on the Microstructure and Adhesion of Zein Coatings to Titanium Substrates

**DOI:** 10.3390/ma14020312

**Published:** 2021-01-09

**Authors:** Filip Maciąg, Tomasz Moskalewicz, Kazimierz Kowalski, Alicja Łukaszczyk, Zoya Hadzhieva, Aldo Roberto Boccaccini

**Affiliations:** 1 Faculty of Metals Engineering and Industrial Computer Science, AGH University of Science and Technology, Czarnowiejska 66, 30-054 Kraków, Poland; maciag@agh.edu.pl (F.M.); kazimierz.kowalski@agh.edu.pl (K.K.); 2 Faculty of Foundry Engineering, AGH University of Science and Technology, Reymonta 23, 30-059 Kraków, Poland; alicjal@agh.edu.pl; 3 Institute of Biomaterials, Department of Materials Science and Engineering, University of Erlangen-Nuremberg, 91058 Erlangen, Germany; zoya.hadzhieva@fau.de (Z.H.); aldo.boccaccini@ww.uni-erlangen.de (A.R.B.)

**Keywords:** zein, coating, electrophoretic deposition, surface topography, titanium

## Abstract

Zein coatings were obtained by electrophoretic deposition (EPD) on commercially pure titanium substrates in an as-received state and after various chemical treatments. The properties of the zein solution, zeta potential and conductivity, at varying pH values were investigated. It was found that the zein content and the ratio of water to ethanol of the solution used for EPD, as well as the process voltage value and time, significantly influence the morphology of coatings. The deposits obtained from the solution containing 150 g/L and 200 g/L of zein and 10 vol % of water and 90 vol % of ethanol, about 4–5 μm thick, were dense and homogeneous. The effect of chemical treatment of the Ti substrate surface prior to EPD on coating adhesion to the substrate was determined. The coatings showed the highest adhesion to the as-received and anodized substrates due to the presence of a thick TiO_2_ layer on their surfaces and the presence of specific surface features. Coated titanium substrates showed slightly lower electrochemical corrosion resistance than the uncoated one in Ringer’s solution. The coatings showed a well-developed surface topography compared to the as-received substrate, and they demonstrated hydrophilic nature. The present results provide new insights for the further development of zein-based composite coatings for biomedical engineering applications.

## 1. Introduction

Titanium and its alloys are currently the most commonly used metallic biomaterials. They are characterized by good biocompatibility, high corrosion resistance, favorable fatigue strength and high strength to weight ratio. In particular, they are used for orthopedic and dental implants because their Young’s modules are relatively low among metallic materials [1,2,3]. Titanium is inert in the human body due to the formation of a passive titanium oxide layer on its surface, but it has weak osteoinductive properties [4,5]. In addition, biofilm formation on titanium implants, which has a decisive influence on the persistence of bacterial infections, is a major problem [6]. Modification of the surface of titanium biomaterials by coatings allows their antibacterial properties, biocompatibility and electrochemical corrosion resistance to be improved [7,8,9,10].

One of the promising biopolymers showing favorable properties as a coating material is zein. It is an alcohol-soluble, natural protein–polymer obtained from corn, which has gained interest in biomedical application due to its biocompatibility, biodegradability and low toxicity [11,12]. Zein is classified in three fractions: α (is the most abundant), β and γ differing in solubility. The major fraction in corn zein is α-zein to the amount of approx. 75–85%, while the β- and γ-zein fractions only make up 10–15% and 5–10%, respectively, depending upon the genotype [12]. The molecular weight of α-zein is within the range of 20–24 ku soluble in 40–95% (*v*/*v*) of ethanol, β-zein has 17–18 ku/30–80% (*v*/*v*) EtOH solubility, while γ-zein has 27 ku/0–80% (*v*/*v*) EtOH. Additionally, γ-zein is soluble in EtOH only in the presence of 2-mercaptoethanol (C_2_H_6_OS) [13]. The typical peak for α-zein on an FTIR spectrum occurs for signals between 1600 and 1700 cm^−1^ [10,14]. The insolubility of zein in water is related to the high content of hydrophobic residues, such as proline, leucine, alanine and phenylalanine [11,15,16]. Zein can be potentially used to deliver drugs inside the cell cytoplasm in pharmaceutical applications, to perform 3D porous zein scaffolds or to form coatings [10,11,16,17,18,19,20]. According to Yong et al. [21], α-zein is composed of highly homologous repeat units and has a high α-helix content.

A convenient method for producing biodegradable polymeric coatings on electrically conductive substrates is electrophoretic deposition (EPD). EPD is an electrochemical method, which has gained interest in biomedical engineering due to the possibility of using a wide range of ceramic and polymeric materials and their combinations during the coating deposit process [22,23,24]. Other advantages of this method are also the short deposition time and relatively simple apparatus. Moreover, the process is usually realized at room temperature, and the coatings can be deposited on substrates with complex shapes. Through adjustment of the applied voltage and deposition time, the morphology and thickness of the coatings can be tailored and are easy to control. In order to deposit homogeneous coatings, it is necessary to use a stable colloidal solution containing charged particles moving when an electric field is applied [22,23,25,26]. Thus, deposition occurs in a colloidal environment, in which the fine particles of the suspended material, the dispersed phase, move in the liquid dispersing phase to the substrate material under the influence of an applied electric field [25]. The concept has been extended to the deposition of molecules from solutions [23].

The EPD of pure zein has not been widely studied in the available literature. Previously, crack-free, well-adhered, robust and transparent pure zein coatings were electrophoretically deposited for the first time on 316L stainless steel substrates by Kaya and Boccaccini [10]. They found that the EPD from an ethanol-based solution with the addition of water enabled the deposition of homogeneous coatings with a smooth surface. Zein coatings containing bioactive glass particles (on stainless steel) were also obtained by Meyer et al. [16].

In this study, we have focused on the EPD of zein on a commercially pure titanium substrate and investigated the selected properties of the obtained coatings. It is well known that the most important property of coating is its good adhesion to the substrate, as well as its homogeneous morphology and microstructure. Thus, the aim of this work was to investigate the influence of the zein solution’s chemical composition and the EPD parameters on the coating morphology and surface topography, as well as the effect of different Ti substrate surface preparation on the coating adhesion to the substrates.

## 2. Material and Methods

**Figure 1:**
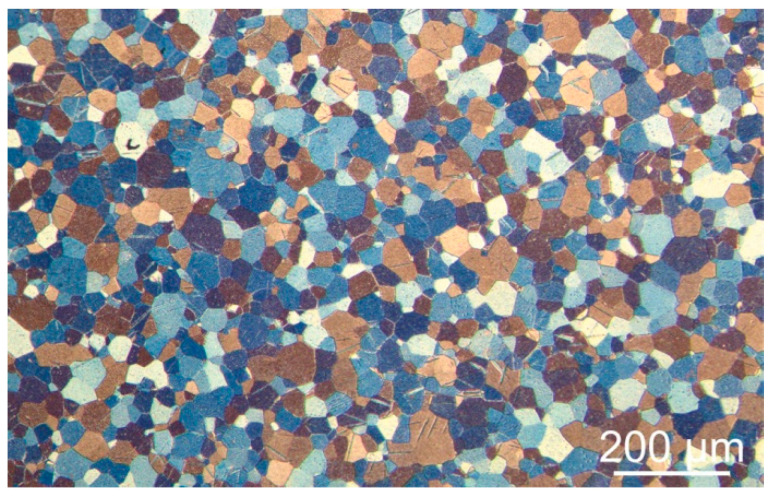
Microstructure of the commercially pure titanium (CP-Ti) Grade 1 substrate, light microscopy (LM) image.

A commercially pure titanium (CP-Ti) Grade 1 sheet, provided by Shanghai Huaxia Industry Co. Ltd., Shanghai, China, was used to prepare substrates for EPD. The microstructure of CP-Ti consisted of α (hexagonal close-packed, hcp) phase grains. The grain equivalent circle diameter (ECD) varied from 5 µm to 90 µm. A typical image of the titanium microstructure, observed with light microscopy (LM) using an OPTA-TECH MM100 instrument (Warsaw, Poland), is shown in Figure 1. Annealing twins were seen to occur in some grains. The CP-Ti samples were in the shape of a cuboid with the dimensions of 35 mm × 15 mm × 0.5 mm. The as-received samples were cleaned with water and degreased with technically pure ethanol prior to deposition. In addition, different chemical treatments, marked as A-D, were used in order to elaborate the coating with good adhesion to the substrates. The chemical treatments were as explained next.

A. The titanium substrates were washed in acetone, soaked in a 0.06 M Na_3_PO_4_*12H_2_O solution at 80 °C, washed in hot water, soaked in a solution of 5 mL hydrogen fluoride (HF) 40 vol %, 35 mL nitric acid (HNO_3_) 70 vol % and 60 mL distilled water for 5 min, washed in water and dried [27].

B. The samples were cleaned with acetone and distilled water in an ultrasonic bath for 5 min, dried and used as a cathode in an electrolytic cell. An aqueous solution of acetic acid (1.5 M) (CH_3_COOH) was used as an electrolyte. The anodic oxidation process was conducted at a voltage of 60 V; the anodic duration was 10 min, at a temperature of ~25 °C. In the end, samples were washed in distilled water, then in acetone and finally dried in laboratory air [28].

C. The substrates were ultrasonically cleaned in acetone and distilled water for 5 min and dried. The Ti substrates were dipped in a solution consisting of 10 mL sulfuric acid (H_2_SO_4_, 95%) and 10 mL perhydrol (H_2_O_2_, 33%) at a temperature of 45 °C for 30 min. After dipping, the samples were washed ultrasonically in distilled water for 30 min and ethanol for 10 min and finally dried in the air [29].

D. Titanium samples were cleaned in an ultrasonic bath using the following reagents: acetone for 15 min, in a 70% EtOH solution for 20 min, and finally in distilled water for 20 min. Then, the samples were etched in solutions composed of 100 mL HCl (18 wt %) and 100 mL H_2_SO_4_ (48 wt %) for 30 min. Subsequently, they were cleaned using distilled water and dried in still air [30].

Zein was obtained in powder form from Sigma-Aldrich, Co. (St. Louis, MO, USA). The components were weighed with the use of an OHAUS CORP model PA214CM/1 scale (Nänikon, Switzerland). All zein solutions (total volume of 50 mL) were prepared by magnetic stirring with an IKA model Ro 5 magnetic stirrer (Staufen, Germany) at room temperature (RT) and then subjected to ultrasonic cleaning with a POLSONIC, Sonic-3 (Warsaw, Poland). The pH values were measured using an ELMETRON CPC-505 pH-meter (Zabrze, Poland). Their chemical composition and pH, as well as the procedure of solution preparation and deposition parameters, are shown in Table 1.

Each solution was prepared in a similar manner. First, a solution of ethanol, distilled water and glycerol was prepared and stirred for 10 min. Then zein powder was gently introduced to the solution. Next, the solutions were subjected to ultrasonic agitation. Finally, the pH was measured, and the deposition took place. The detailed parameters of solution preparation and parameters used for EPD are given in Table 2.

The zeta potential and the conductivity of the zein solution were studied by laser Doppler velocimetry (LDV) technique. The Malvern Zetasizer Nanodevice (Malvern Instruments Ltd., (Malvern, UK) was used for measurements. In order to obtain reliable and representative results, 0.1 g/L zein was diluted in 90 vol % ethanol and 10 vol % water. Zeta potential values were obtained based on the Debye–Hückel equation. The pH value of the solution was adjusted by using acetic acid or NaOH (0.5 M).

The deposition was performed in a two-electrode cell. An austenitic stainless steel plate (AISI 316L) served as a counter electrode. During the deposition process, the electrodes were kept at a constant distance of 10 mm. An EX752M Multimode PSU (AIM-TTI, Huntingdon, UK) power supply was used during the deposition. The effect of the chemical composition of the zein solution, applied voltage and deposition time on current density during the EPD process was investigated using a Tektronix DMM 4040 Multimeter (TEKTRONIX, Oldbury, Bracknell, Berkshire, UK).

The morphology of the coatings was investigated by LM and a scanning electron microscope (SEM) FEI Nova NanoSEM450 (FEI, Eindhoven, the Netherlands). The coatings microstructure was investigated using a transmission electron microscope (TEM), JEOL JEM-2010 ARP (JEOL, Tokyo, Japan). Thin foils made of EPD zein solutions for TEM investigation were prepared by placing a drop of the zein solution on a copper grid covered with carbon film and dried. The cross-sectional slice (lamella) for the TEM investigation of the coating was prepared by a focus ion beam (FIB) milling using an FEI QUANTA 3D 200i device, FEI, Eindhoven, The Netherlands. Phase identification of the coated titanium was performed by Grazing Incidence X-ray diffractometry (GIXRD), with the use of a Panalytical Empyrean DY1061 X-ray diffractometer, Malvern Panalytical, Almelo, the Netherlands.

The topography of the substrate and the coating surface was examined by a Veeco WYKO NT930 optical profilometer (Veeco, Plainview, NY, USA). The oxidation state of titanium of the differently treated titanium plates was investigated using the X-ray hotoelectron spectroscopy (XPS) method. An instrument of Vacuum Systems Workshop operating with the K_α_ Mg radiation of energy 1253.6 eV was used. A hemispherical analyzer worked in a fixed-analyze transmission mode (FAT) with a constant pass energy of electrons set on 22.5 eV. The binding energy scale of XPS spectra was calibrated by fixing the position of the dominant C 1 s peak of the adventitious carbon to 284.6 eV. During the analyses, the residual pressure in the vacuum chamber was lower than 5 × 10^−8^ mbar.

The contact angle (CA) and surface free energy (SFE) of the substrate and zein coating were examined by a Krüss DSA25E goniometer (Hamburg, Germany). During the measurement, water (H_2_O) as a polar liquid and diiodomethane (CH_2_I_2_) as a nonpolar liquid were used. Values of the total SFE, as well as its polar and dispersive components, were calculated using the Owens–Wendt–Rabel–Kaelble (OWRK) method.

A tape test was conducted to investigate the adhesion of coatings to the underlying titanium substrate. The measurement was carried out using the cross-cut adhesion test method according to the ASTM D3359-17 standard [31]. The method involved making two perpendicular incisions 20–30 mm long at an angle of 90 degrees, using a knife equipped with 6 blades. Then, a standardized tape was glued on and, after it was peeled off, the coating adhesion to the underlying substrate was characterized in accordance with the standard.

Electrochemical investigations were carried out in the Ringer’s solution at a temperature of 37 °C. The composition of Ringer’s solution was as follows: 8.6 g/L NaCl, 0.3 g/L KCl, 0.25 g/L CaCl_2_ in distilled water. The pH of this solution was 7.4. The Ringer’s solution was de-aerated throughout the tests. A PGSTAT302 Autolab potentiostat/galvanostat module (Utrecht, the Netherlands) was used in these experiments. All potentials were measured vs. SCE (3 M KCl solution), and the counter electrode was made of platinum wire. Electrochemical measurements were performed using a classical three-electrode cell. Upon immersion of the specimens into the electrolyte, the open-circuit potential was measured as a function of time until a stable value was reached. The polarization curves were obtained with a scan rate of 1 mV/s. The corrosion rate was defined by limiting current density, which passes through the passivating film [32].

## 3. Results and Discussion

### 3.1. Optimization of Zein Solution Composition and EPD Parameters

**Figure 2:**
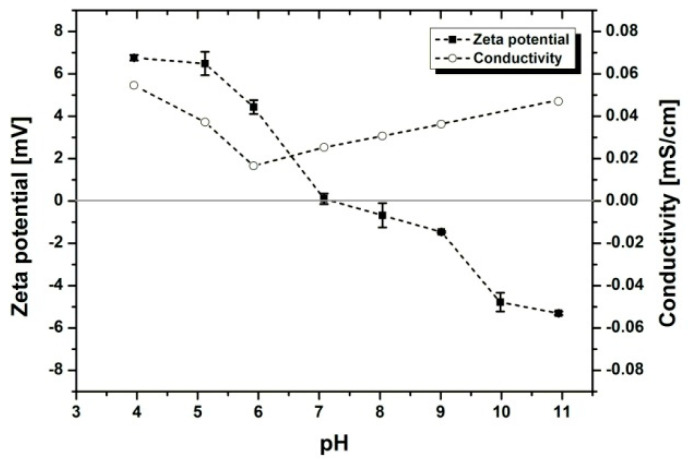
Zeta potential and conductivity of zein-ethanol-water solution at varying pHs.

Stable colloidal solutions are usually developed based on their high zeta potential. Both positive and negative charges should be considered [33]. Another key factor to take into account for successful EPD is the conductivity of the solution [22]. Previous studies have revealed that if the electrical conductivity of a suspension is too high, the particle motion could be inhibited, thereby reducing the deposition growth [34]. Nevertheless, if the suspension is too resistive, the particles charge electronically, and the stability is diminished [35]. As shown in Figure 2, with decreasing pH, the zeta potential of the zein solution increases. This implies that in acidic conditions, the glutamine amide groups in the zein structure protonate, leading to stronger electrostatic repulsion forces between the polymer chains [36]. The isoelectric point of zein lies in the neutral range, which is in good agreement with the literature [37]. In alkaline solutions, more negative charges are introduced to the protein structure due to the hydrolysis of glutamine to glutamate and the deprotonation of carboxylic groups on glutamate [36,38]. It was observed that the conductivity of the solution was minimal near the initial pH (around 5.7) and maximal in highly acidic and basic environments.

On one hand, the high conductivity at low pH can be explained by the increased degree of ionization of the zein amino group [39]. Moreover, the use of external additives such as acids and bases for pH adjustments could lead to the accumulation of an excess number of free ions in the solution [40]. Consequently, these ions may act as major charge carriers, reducing the electrophoretic mobility of the polymer molecules and deteriorating the quality of the deposited coating [22]. Therefore, in order to achieve stable solutions with high zeta potential and low conductivity values, no further pH adjustments of the EPD solutions were performed in this work.

The zeta potential values of zein–ethanol–water EPD solutions at varying ethanol concentrations are shown in Table 3. It was observed that with increasing ethanol content of the solvent, the zeta potential also increased. These results can be explained by the amphiphilic nature of zein molecules and their ability to form macromolecular micelle-like structures when solvated in ethanol. Previous studies indicated that as the ethanol concentration increased from 70% to 90%, the aggregation number of zein molecules significantly decreased [41,42,43]. According to Kim et al. [41], at ethanol concentrations below 90%, the aggregation forces orientate the hydrophobic moieties of zein molecule toward the center of the micelle structure and the hydrophilic residues toward the solvent medium. In this case, the surface charges of the molecules repel each other, whereby resulting in high solution stability. As the ethanol content increases above 90%, an inversion of the micelle-like structure occurs, leading to electrostatic attractions of the zein molecules and subsequent aggregation and precipitation. Thus, the measured zeta potential values confirm that the solution containing 90 vol % of ethanol is the most stable one, which is an important prerequisite to obtain high-quality EPD coatings.

**Figure 3:**
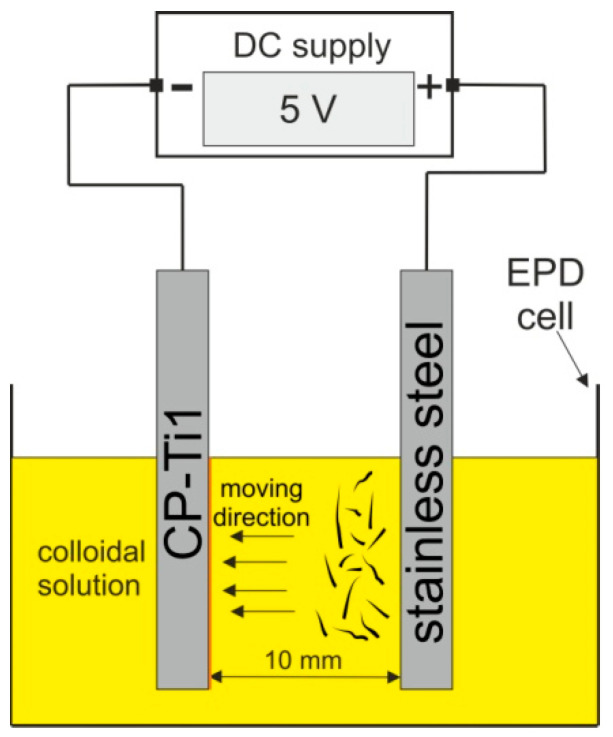
Scheme of the mechanism of EPD of zein.

Kaya and Boccaccini [10] have proposed a mechanism for zein deposition during EPD. Positively charged zein particles under the influence of an applied electric field can move towards the cathode. During the EPD process in aqueous solutions, a reduction of water occurs on the cathode, causing an increase in the pH at its surface [44]. Watson et al. [45] reported that the isoelectric point of zein is in the pH range 5–6, and the critical precipitation limit was in the pH range 4–5. It was speculated that this effect explains the electrophoretic movement of zein molecules to the cathode, and following neutralization of protonated zein macromolecules in the area of increased pH on the cathode surface, zein deposition is obtained [10]. Figure 3 shows a simplified scheme of the EPD of zein.

The chemical composition of the zein solution for the EPD of coatings was elaborated based on an experimental, trial-and-error method. It was reported in the literature [10,46] that zein coatings deposited from an aqueous ethanol solution have poor mechanical properties, which makes them prone to cracking. Therefore, in the present work, a plasticizer in the form of glycerol was added to the solution to improve the mechanical stability of coatings.

**Figure 4:**
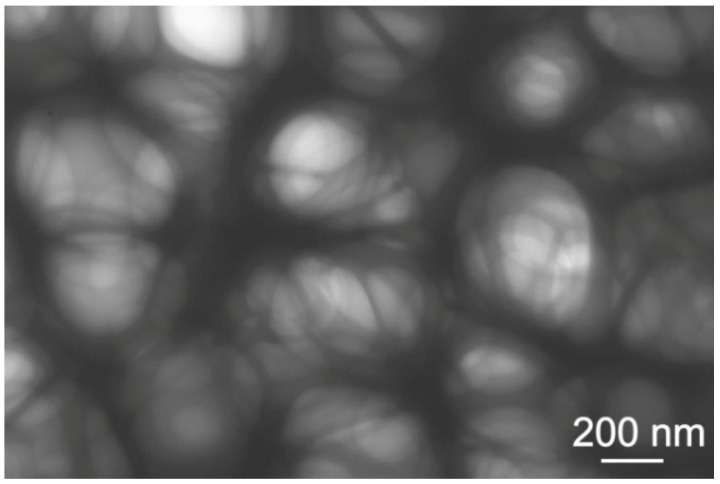
TEM image of the colloidal solution consisting of 90 vol % of ethanol, 10 vol % of distilled water, 20 wt % of glycerol and 150 g/L of zein.

Observation of a solution composed of 90 vol % ethanol, 10 vol % distilled water, 20 wt % glycerol and 150 g/L zein performed by TEM showed that it consisted of tangled chains of up to several micrometers in length (Figure 4).

**Figure 5:**
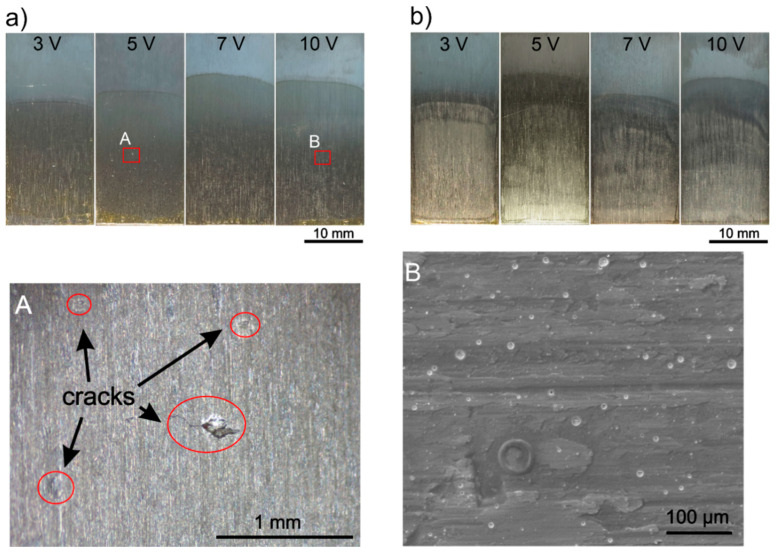
Macroscopic images of CP-Ti samples with a zein coating deposited from a solution containing 85 vol % (**a**) and 75 vol % (**b**) of ethanol, distilled water (balance), 20 wt % of glycerol and 150 g/L of zein at different voltage values and a deposition time of 5 min. Stereoscopic microscope (**A**) and SEM (**B**) images of magnified details of the coating deposited from a solution containing 85 vol % at 5 V and 10 V, respectively, are also shown in the lower part of the figure.

EPD was carried out with the use of several solutions with various chemical compositions (Table 1) at different deposition parameters, voltage and time (Table 2) to optimize the process and to obtain macroscopically homogeneous zein coatings. The number of prepared solutions allowed the influence of chemical composition and deposition parameters on the coating quality to be observed. The homogeneity of the obtained coatings was assessed on the basis of macroscopic and microscopic observations. Figure 5a,b show macroscopic images of CP-Ti samples with a zein coating deposited from a solution containing 85 vol % (Figure 5a) and 75 vol % (Figure 5b) of ethanol, distilled water (balance), 20 wt % of glycerol and 150 g/L of zein at different voltage values of 3 V, 5 V, 7 V and 10 V as well as constant deposition time of 5 min.

It was found that all coatings were nonhomogeneous with the presence of microcracks (Figure 5, image A) and a high number of open micropores with diameters of up to 20 μm (Figure 5, image B). Nonhomogeneity of the coatings can be explained by a decrease of pH values (Table 1) and zeta potential of the solution (Table 3) caused by an increase in the volume of distilled water in the solution, e.g., from 10 vol % to 15 vol % or 25 vol %.

**Figure 6:**
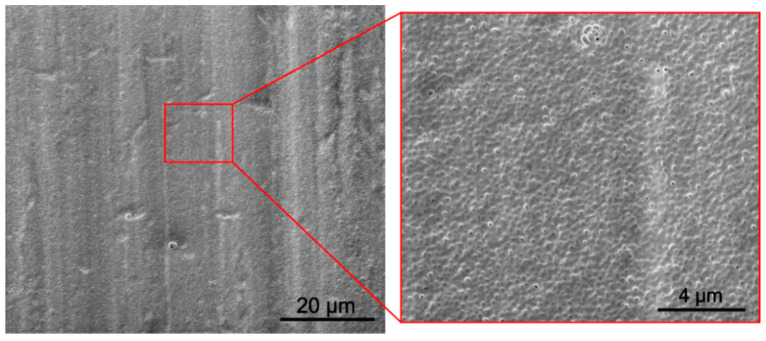
SEM images of the zein coating deposited from a solution containing 90 vol % of ethanol, 10 vol % of distilled water, 20 wt % of glycerol and 100 g/L of zein at 7 V for 5 min.

Higher quality coatings were obtained from solutions containing 90 vol % of ethanol and 10 vol % of distilled water. However, in this case, the content of zein influenced the coating homogeneity. A coating deposited from the solution containing 100 g/L zein (solution no 1 in Table 1) was very thin, exhibited nonuniform thickness and a presence of a high number of open pores on the surface (Figure 6).

**Figure 7:**
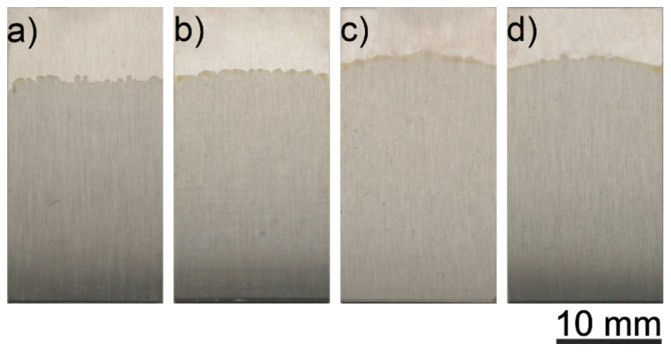
CP-Ti samples with a zein coating deposited from a solution containing 90 vol % of ethanol, 10 vol % of distilled water, 20 wt % of glycerol and 150 g/L of zein at different voltage values 3 V (**a**), 5 V (**b**), 7 V (**c**), 10 V (**d**) and a deposition time of 5 min.

The most promising coatings were deposited from the solutions numbered 3 and 6 (Table 1), i.e., consisting of 90 vol % ethanol, 10 vol % distilled water, 20 wt % glycerol and 150 g/L zein for solution 3 and 200 g/L of zein for solution 6. The macroscopic images of the coatings deposited from the solution containing 150 g/L zein in an EtOH/distilled water mixture with the volume ratio of 90/10 at a different voltage of 3 V, 5 V, 7 V and 10 V, as well as 5 min deposition time, are shown in Figure 7.

**Figure 8:**
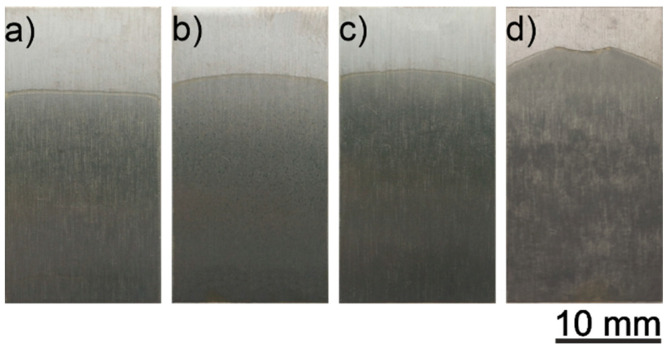
CP-Ti samples with a zein coating deposited from a solution containing 90 vol % of ethanol, 10 vol % of distilled water, 20 wt % of glycerol and 200 g/L of zein at voltages 3 V (**a**), 5 V (**b**), 7 V (**c**) and 10 V (**d**) and a deposition time of 5 min.

All coatings deposited on titanium substrates were uniform and transparent. They were very similar to each other macroscopically. Voltages in the range 3–10 V did not noticeably affect the quality of the coatings. Figure 8 presents the macroscopic images of the coatings deposited from a solution containing 200 g/L of zein in an EtOH/distilled water mixed with a volume ratio of 90/10 at different voltages of 3 V, 5 V, 7 V and 10 V, and 5 min deposition time.

Similar to the coatings deposited from the zein solution containing 150 g/L of zein, these coatings were uniform and transparent, regardless of the voltage value used for deposition. Thus, the voltage of 5 V was selected for the deposition of final coatings.

**Figure 9:**
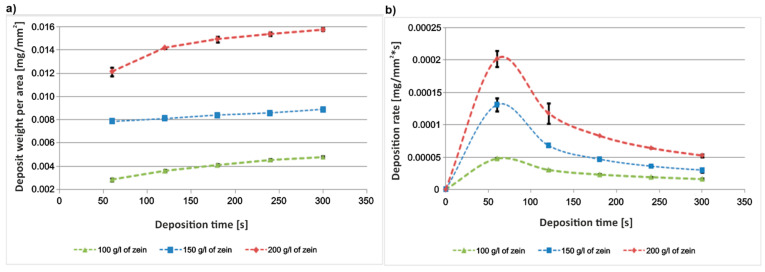
Weight change (**a**) and deposition rate (**b**) of as-deposited zein coatings at a constant voltage of 5 V and deposition time of 300 s for EPD from the solutions with different zein concentrations of 100 g/L, 150 g/L and 200 g/L.

**Figure 10:**
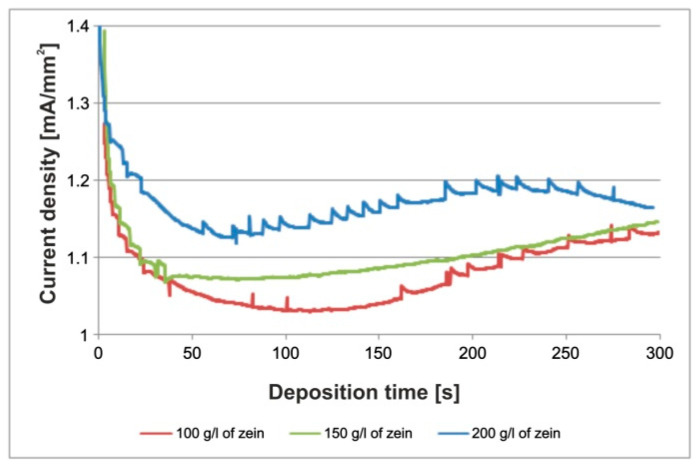
Dependence of current density and deposition time during the deposition process for different zein contents (100 g/L, 150 g/L and 200 g/L) in an EtOH/distilled water solution with a volume ratio of 90/10 vol % at a voltage of 5 V.

It was also observed that voltages higher than 7 V led to the appearance of an increasing number of bubbles in the solution and intense oxidation of the substrate. It is well known [47] that during EPD from aqueous solutions, electrolysis of water occurs. As a result of this reaction, under the influence of an applied external voltage, water decomposes into oxygen and hydrogen, forming bubbles on the electrode surface. Increasing the volume fraction of water in the solution resulted in an increase in electrolysis. The variation of the mass of the coating with deposition time was also examined. In the case of coatings obtained from a solution containing 100 g/L and 150 g/L of zein, the coating weight increased almost linearly (Figure 9a). The weight of coatings deposited from solutions containing 200 g/L of zein increase rapidly in the first two minutes of deposition, and then this increase stabilized and proceeded in a nearly linear manner (Figure 9a). However, in the case of all solutions, the weight gain was very small, in the order of ten-thousandths of a gram. The deposition rate for all EPD processes was very similar, i.e., a rapid increase at the beginning of the deposition process followed by a gradual decrease in the rate of deposition until the end of the process (Figure 9b). The current density during EPD for all solutions rapidly decreased in the first minute and then stabilized and slightly increased, however at the end of the process, it began to fall again in the case of EPD from the solutions containing 100 g/L and 200 g/L of zein (Figure 10). The higher turbulence of the current density for the 100 g/L and 200 g/L zein solutions indicates vigorous bubbling occurring during EPD. This contributed to the formation of a higher number of pores on the coating surface compared to coatings obtained from a solution containing 150 g/L zein (see below).

Summarizing, the chemical composition of the solution, both zein content and water/ethanol ratio, as well as the voltage used during EPD, were the most important factors that affected the coating homogeneity. It was found that the low zein content in the solution, e.g., 100 g/L, resulted in the formation of thin coatings with non-equal thickness and a high number of open pores. It is believed that this could be a result of the higher pH value of the solution compared with the pH values of solutions with the addition of 150 g/L or 200 g/L zein (Table 1). In turn, the higher pH leads to an increase of conductivity and a decrease of the zeta potential of the solution (Figure 2). On the other hand, the content of zein in the solution greater than 200 g/L caused high solution density, which makes EPD difficult. Similarly, increasing the water content from 10 vol % to 15 vol % or 25 vol % in the solution resulted in obtaining nonhomogeneous coatings due to the low electrophoretic mobility of zein molecules and chains (Table 3). Moreover, both the water content in the solution greater than 10% and a deposition voltage greater than 7 V lead to an increase in water electrolysis, resulting in the formation of gas bubbles in the solution and finally pores in the coating.

### 3.2. Adhesion of the Coating to the Titanium Substrates

**Figure 11:**
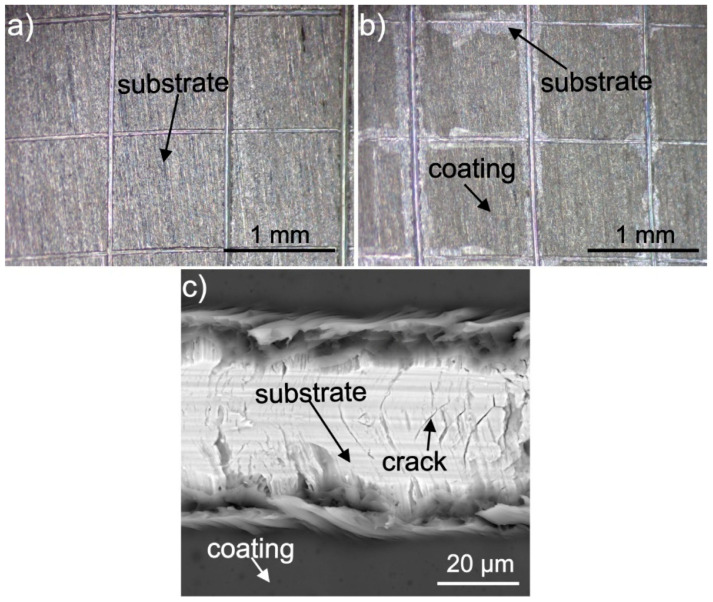
Stereoscopic microscope (**a**,**b**) and SEM (**c**) images of as-received titanium coated with zein after the tape test. The coatings were deposited from a solution containing 90 vol % ethanol, 10 vol % distilled water, 20 wt % glycerol and 150 g/L (**a**) or 200 g/L (**b**,**c**) of zein.

The tape tests revealed that the coatings obtained from the solution consisting of 90 vol % ethanol, 10 vol % distilled water, 20 wt % glycerol, and 150 g/L zein had very poor adhesion to the as-received CP-Ti substrate, while the coatings obtained from the solution containing 90 vol % ethanol, 10 vol % distilled water, 20 wt % glycerol and 200 g/L zein had significantly greater adhesion to the as-received substrate. The surfaces of both samples after the test are shown in Figure 11. The coating deposited from the solution containing 150 g/L (Table 1, solution 3) of zein was completely removed from the substrate (Figure 11a), while the coating containing 200 g/L zein (Table 1, solution 6) was removed only from the areas close to the cross-cuts (Figure 11b). According to ASTM D3359-17, the adhesion class for the coating deposited from solution 3 was 0B (more than 65% of coatings area removed) and 4B for coating obtained from solution 6 (under 5% of the coating area removed). It is speculated that such behavior may result from different coating thickness and different glycerol content in the coating. In comparison, Kaya et al. reported a coating adhesion of 5B for first cycle EPD coatings deposited from 150 g/L zein solutions on stainless steel substrates [10]. 

Nevertheless, EPD was performed at a longer deposition time (10 min) and higher voltage (10 V), which could be the reason for the differences in the adhesion strengths of the zein coatings obtained. Detailed investigation of the surface of the sample with a coating deposited from solution 6 after the tape test using SEM led to the observation that the coating was detached from the substrate in the cross-cut during the test (Figure 11c). In addition, cohesive cracks perpendicular to the direction of the incision appeared inside the cuts in the oxide layer covering the substrate under the influence of a load of the cutting tool. Zein was rubbed into them during the test.

It is well known that the substrate surface preparation influences the coating adhesion to the substrate. Therefore, in this work, the effect of substrate preparation on coating adhesion was also investigated. Coatings obtained from a solution containing 200 g/L of zein were deposited on the chemically treated substrates (according to procedures A–D described in the Experimental section) at a constant voltage of 5 V and a deposition time of 5 min. In addition, for the B–D treatments, the deposition time was shortened to investigate the effect of coating thickness on the coating adhesion to the substrate.

**Figure 12:**
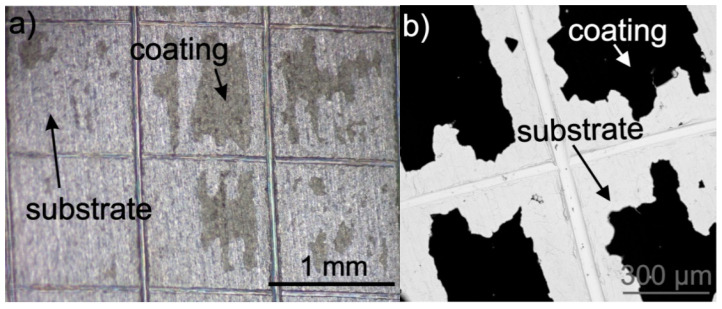
Stereoscopic microscope (**a**) and SEM (**b**) images of titanium were chemically treated according to procedure A and coated with zein after the tape test. The coating was deposited from a solution containing 90 vol % ethanol, 10 vol % distilled water, 20 wt % glycerol and 200 g/L zein, at a voltage of 5 V and deposition time 5 min.

The adhesion of coatings to the substrate chemically treated in an acid solution of HF, HNO_3_ and distilled water (treatment A) was significantly lower than that on the as-received sample (Figure 12). Stereoscopic microscope and SEM observations indicated that the adhesion class, in this case, was 0B (according to ASTM D3359-17). Figure 12 shows that the coating was not completely detached from the titanium substrate.

**Figure 13:**
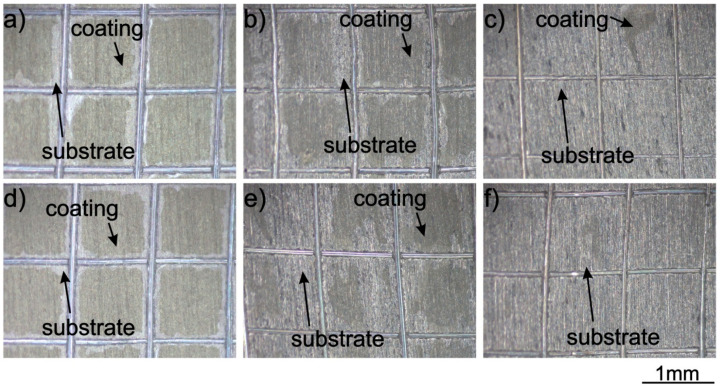
Stereoscopic microscope images of titanium were chemically treated according to procedures B–D and coated with zein after the tape test. The coating was deposited from solution 6 at a voltage of 5 V and a deposition time of 2.5 (**a**–**c**) and 5 (**d**–**f**) minutes.

Anodizing (treatment B) was carried out for deposition times of 2.5 and 5 min and gave similar results to the as-received sample, i.e., the adhesion class of the coating was also 4B for both deposition times (Figure 13a,d). The adhesion of the coating to the substrate after etching in piranha solution (treatment C) was visibly weaker, and the adhesion class was 2B (Figure 13b,e). Etching of the substrate in a mixture of H_2_SO_4_ and HCl (treatment D) did not increase the coating adhesion, and the class 0B was determined (Figure 13c,f).

Table 4 summarizes the adhesion class of zein coatings to the as-received CP-Ti1 substrate and to substrates after various A–D chemical treatments.

**Figure 14:**
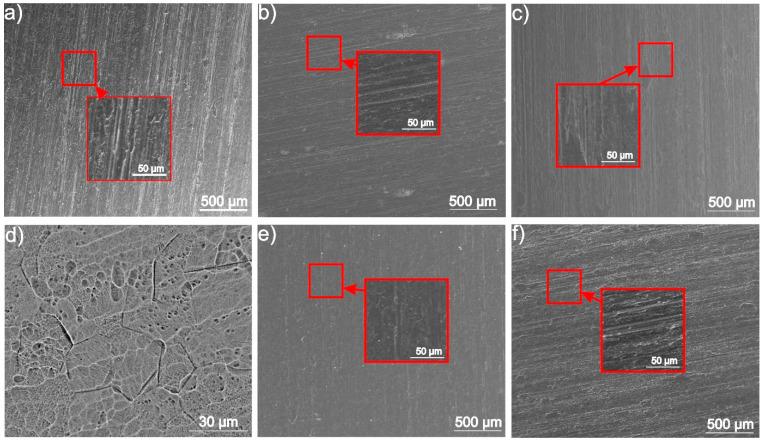
SEM morphology of the as-received CP-Ti1 (**a**) and chemically treated samples according to procedures A (**b**), B (**c**,**d**), C (**e**) and D (**f**).

To explain the differences of coating adhesion to the as-received and chemically treated substrates, surface morphology and roughness, as well as surface chemistry, were investigated. The morphology of the as-received and chemically treated titanium substrates are shown in Figure 14.

**Figure 15:**
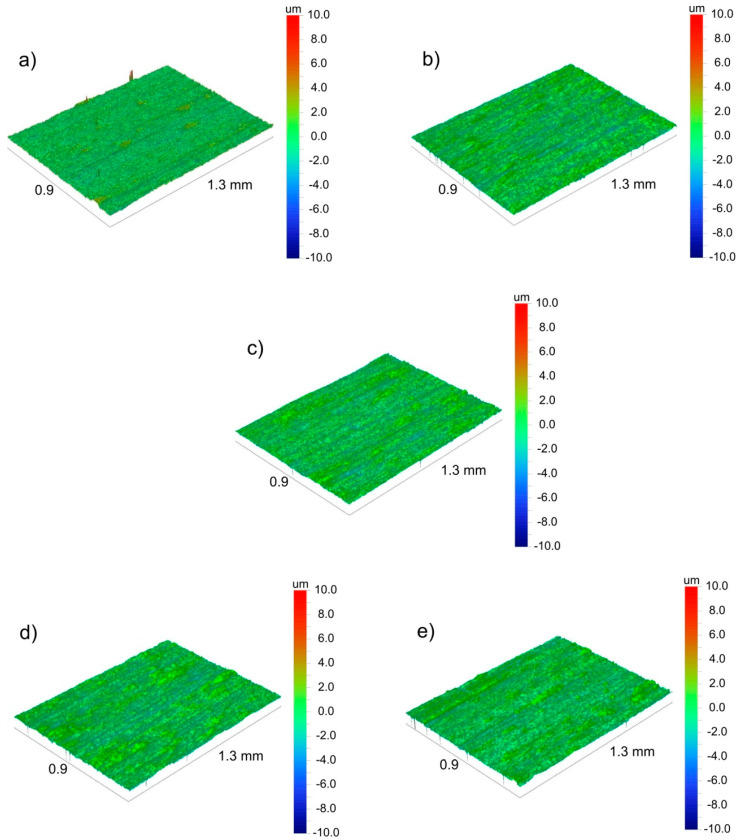
Surface topography of the as-received CP-Ti1 (**a**) and chemically treated substrates according to procedures A-D (**b**–**e**), respectively, observed by optical profilometry.

The SEM observations indicate that relatively deep and clearly visible scratches occurred in the as-received titanium surface (Figure 14a). The disappearance of scratches after chemical treatments associated with etching can be noticed (Figure 14b–f). It was observed that the anodic oxidation (treatment B) caused the formation of open pores in the surface oxide layer covering the titanium substrate (Figure 14d). The surface topography images of all investigated titanium substrates are shown in Figure 15. The surface roughness parameters, the mean square roughness (Rq), the average roughness (Ra) and the total roughness (Rt) are presented in Table 5. Interestingly, the highest surface development was found for chemically treated samples. The highest development of surface topography among them exhibited the sample after treatment B, i.e., after anodic oxidation (Figure 15c) due to the presence of open porosity in the surface oxide layer (Figure 14d). Nevertheless, the scratches were the deepest in sample A (Figure 15b), which is in agreement with the SEM observations.

**Figure 16:**
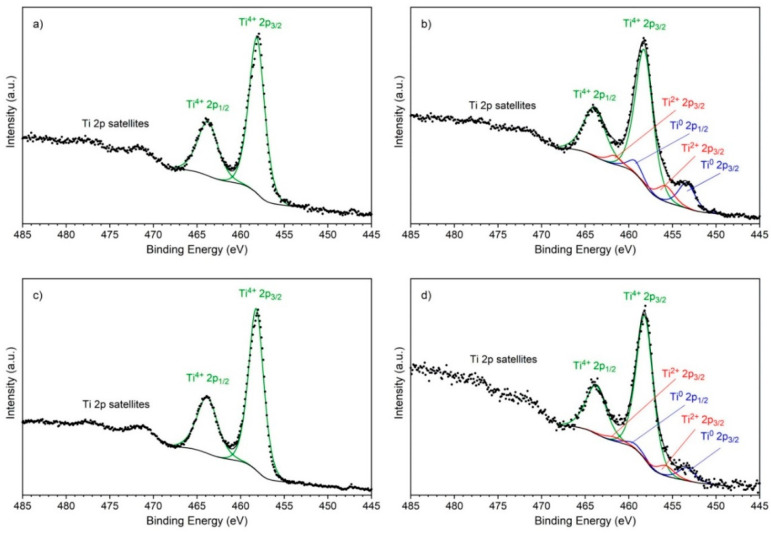
XPS spectra of the Ti 2p line of the as-received (**a**) and chemically treated samples according to procedure A (**b**), B (**c**) and C (**d**).

The surface chemistry of the surface of the titanium substrates was investigated by XPS. The spectra of the Ti 2p line are shown in Figure 16.

Spectra of the as-received sample (Figure 16a) and the sample after treatment B (Figure 16c) showed only one oxidation state of titanium +4 as evidenced by the position of the Ti 2p_3/2_ line on the binding energy scale equal to 458.2 eV. The lack of a line corresponding to the zeroth oxidation (metallic) state additionally proved that the thickness of the surface oxide was greater than a dozen nanometers, as this is the sampling depth of the XPS method in the case of oxides. Spectra of the samples after treatment A (Figure 16b) and after treatment C (Figure 16d) exhibited three oxidation states of titanium +4, +2 and 0. The position of spectral lines Ti 2p_3/2_ corresponding to the +2 oxidation state are equal to 455.8 eV, and corresponding to 0 oxidation state is equal to 453.4 eV. This time a presence of the spectral lines corresponding to the metallic state indicated that the depth of the surface oxide layers on these two samples is clearly thinner than that of the as-received sample and the sample after treatment B. The oxide layer thickness of the sample after treatment A is smaller than that of the sample after treatment C, which is proved by a stronger ratio of the intensities of the spectral lines corresponding to the metallic and oxide states of titanium.

The investigation results obtained from SEM, optical profilometry and XPS indicate that the surface topography, including roughness and morphology, as well as surface chemistry have an effect on the coating adhesion to titanium substrates. However, taking into account only slightly different roughness parameters (R_a_ and R_q_) in all samples and the low adhesion of the coating to substrates A, C and D, it can be assumed that in this specific case, surface features and surface chemistry may be of greater importance. In the case of the as-received substrate, deep scratches were present on the surface (R_max_ = 22.8 ± 5.7 μm). In the case of the substrate after treatment B, the scratches were shallower (R_max_ = 10.8 ± 1.7 μm), but the open porosity occurred on the surface (Figure 14d). Moreover, in this sample, the values of R_a_ and R_q_ were the highest. Thus, due to mechanical interlocking, the coating may have a higher class of adhesion to these substrates than to the samples treated according to procedures A, C and D, for which the R_max_ was lower and open porosity was not observed. On the other hand, in both, as received and chemically treated (B) samples, only one oxidation state of titanium (+4), as evidenced by the position of the Ti 2p_3/2_ line, was found on the surface. Therefore, it can be assumed that the presence of a relatively thick TiO_2_ layer enhanced the adhesion of zein to titanium substrates, which indicates that the chemical bonding may occur between the zein and TiO_2_ layer. To summarize, the combination of both adhesion mechanisms, mechanical interlocking and chemical bonding, may contribute to obtaining high adhesion of zein to titanium substrates.

The coatings deposited from zein solution no 6 on the as-received substrates were selected for further investigation of microstructure and properties due to the highest adhesion achieved on this type of substrates. A great advantage in this particular case is the possibility of using titanium substrates without additional chemical treatment.

### 3.3. Morphology, Microstructure and Surface Topography of Coatings

**Figure 17:**
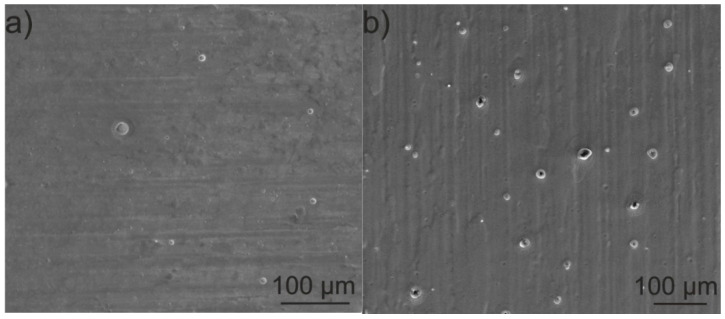
SEM images showing the morphology of zein coatings on CP-Ti1 deposited from solution 3 containing 150 g/L of zein (**a**) and solution 6 containing 200 g/L of zein (**b**) at a voltage of 5 V and deposition time of 5 min.

The SEM investigation revealed that coatings deposited on the as-received titanium from both zein solutions (3 and 6, Table 1) at a voltage of 5 V for 5 min were dense and relatively homogeneous (Figure 17a,b). However, in both coatings, open pores with a diameter in the range of 1–25 µm were observed.

The presence of pores is the result of the electrolysis of water that occurs during the EPD process [47]. The number of pores in the coating deposited from the solution containing 200 g/L zein was higher than in the coating deposited from the solution containing 150 g/L of zein. It is possible that this is due to the higher density and viscosity of the solution containing the higher zein content. It was observed during EPD that, as a result of electrolysis, bubbles appear at the solution/substrate interface.

**Figure 18:**
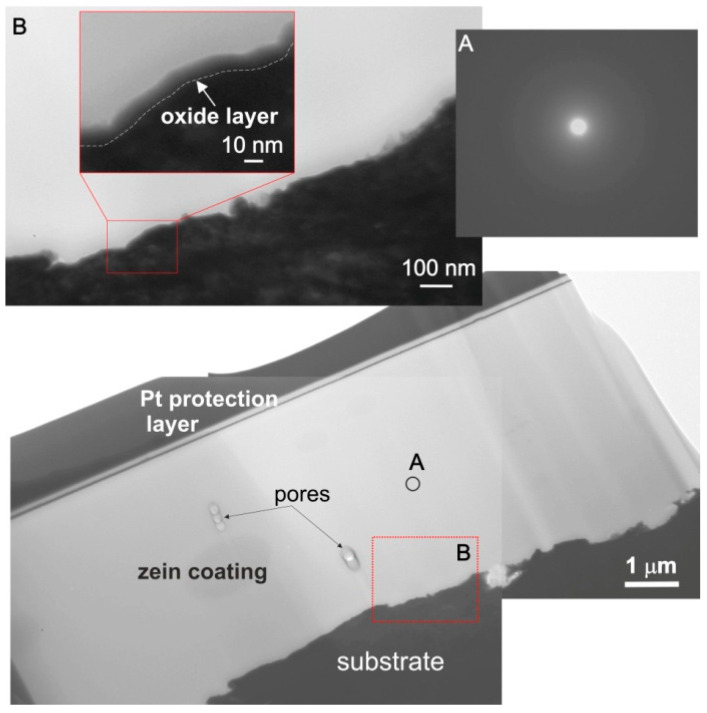
Microstructure (TEM) of the zein coating deposited from solution no 6 on CP-Ti1 and the electron diffraction pattern taken from the zone marked with letter A. Enlarged details of the zone marked with letter B are shown at the top of the figure.

The microstructure was investigated by TEM (Figure 18) on lamella prepared by FIB from a cross-section of the coating deposited from solution 6. The coating thickness was about 4.4 µm.

**Figure 19:**
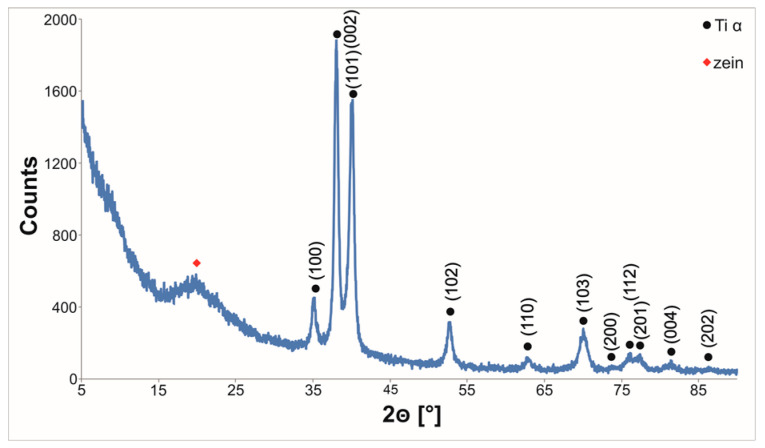
GIXRD pattern of the zein coating deposited on the as-received CP-Ti1 from solution 6 by EPD at a voltage of 5 V, for 5 min. The pattern was obtained at a low incidence angle of 1°.

The microstructure of the coating was dense. The electron diffraction pattern taken from the coating contained diffused rings indicating its amorphous structure. Sporadically, closed pores with a diameter in the range of 1–10 µm were found only in the middle part of the coating. Therefore, it can be assumed that during EPD, gas bubbles (H_2_) move to the surface of the coating. It can be observed from Figure 18 (inset B) that between the coating and the titanium substrate, an oxide layer, about ∼10 nm thick, was present. Closed pores were not observed directly on the oxide/substrate surface. The phase composition of the coated titanium was investigated by grazing incidence X-ray diffractometry. A broad peak at 2θ equal to 15–25° occurred in the GIXRD pattern confirmed the presence of an amorphous zein phase in the coating (Figure 19). The high-intensity diffraction peaks of the Ti α (hcp) phase in the pattern confirmed the low coating thickness.

**Figure 20:**
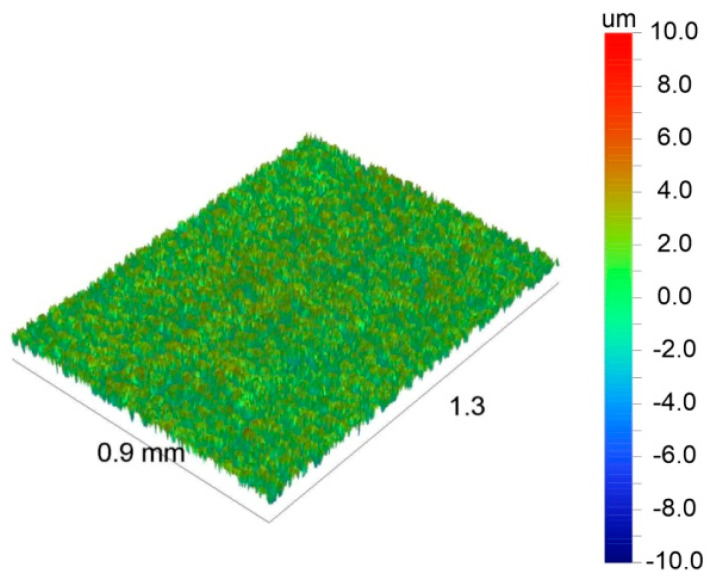
Surface topography of the zein coating (200 g/l) was observed by optical profilometry.

The coating deposited from solution no 6 containing 200 g/L of zein was selected for surface topography characterization. It was found that the coating showed well-developed surface topography in comparison to the as-received titanium substrate (Figure 20). The surface topography parameters of the coating determined from optical profilometry images were as follows: R_a_ = 1.5 ± 0.1 μm, R_q_ = 1.7 ± 0.1 μm and R_max_ = 12 ± 10 μm.

### 3.4. Wettability

**Figure 21:**
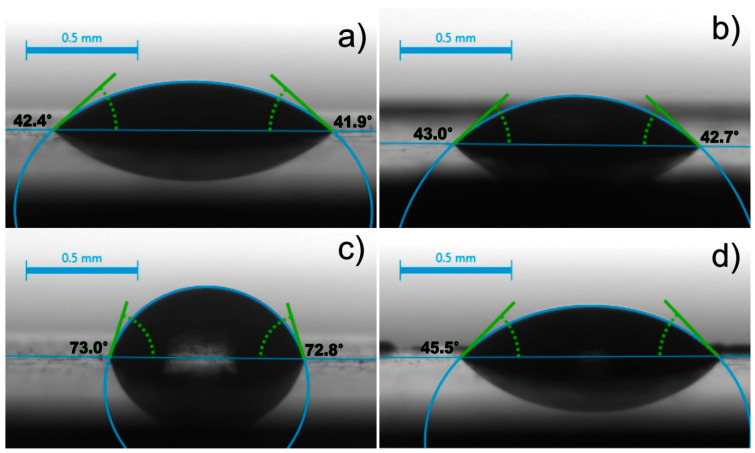
Representative camera images obtained during the measurement of the wettability, showing drops of diiodomethane on the surface of the as-received titanium (**a**) and the zein coating (200 g/L) (**b**) as well as drops of water on the surface of the as-received titanium (**c**) and the zein coating (200 g/L) (**d**).

The wettability of a coating is important in biomedical applications as it gives an insight into the interaction of proteins and cells with the surface [48]. To show the difference in wettability before and after deposition, this parameter was also measured on the as-received substrate. The measurements of the contact angle (CA) and surface free energy (SFE) were carried out using the static sessile drop method. Both water (H_2_O) as a polar liquid and diiodomethane (CH_2_I_2_) as a nonpolar liquid were used as liquid media due to the high surface tension value. Figure 21 demonstrates the average diiodomethane contact angle and water contact angle of the uncoated as-received titanium and zein coated as-received titanium. The mean CA value for the as-received substrate was 43.4 ± 1.6° for diiodomethane (Figure 21a) and 75.6 ± 2.7° for water (Figure 21c). Meanwhile, the mean CA values for the zein coating deposited from solution 6 was 43.5 ± 2.8° for diiodomethane (Figure 21b) and 45.3 ± 3.8° for water (Figure 21d).

These results suggest that both polar and nonpolar liquids can easily wet the coating surface and confirm the presence of polar (e.g., glutamine) and nonpolar (e.g., proline, leucine, alanine) amino acid residues in zein [49]. The SFE for the coating (59.5 ± 3.8 mN/m) was higher than the SFE for the substrate (43.4 ± 2.0 mN/m). The substrate and coating were (weakly) hydrophilic [50]. The hydrophilicity of zein coatings is beneficial for decreasing platelet adhesion and protein adsorption and, thus, for avoiding blood thrombosis or bacterial infection upon implantation in the human body [51,52]. In addition, the hydrophilic character of zein coatings is expected to favor cell attachment, spreading, proliferation, and differentiation [53].

### 3.5. Electrochemical Corrosion Resistance

**Figure 22:**
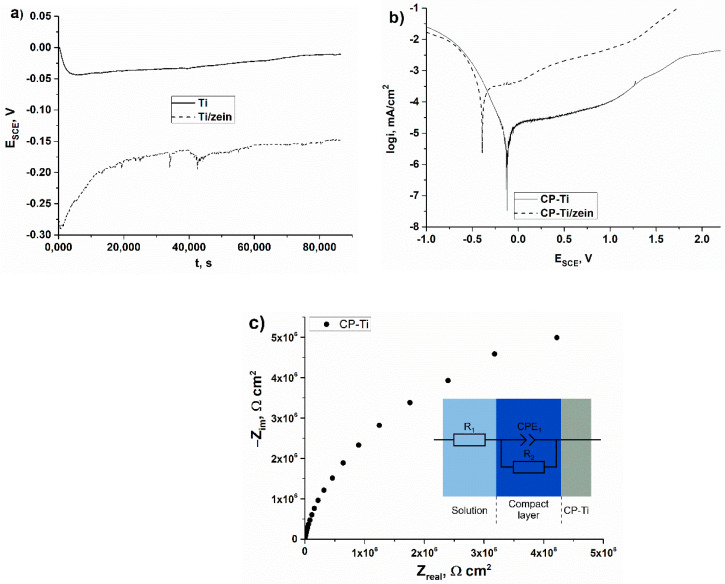
Effect of time on the corrosion potential (**a**) and polarization curves obtained with a scan rate of 1 mV/s (**b**) for as-received titanium and as-received titanium coated with zein as well as a Nyquist impedance plot, equivalent circuit and the scheme of the formation of an oxide layer (**c**). Measurements were performed in Ringer’s solution at a constant temperature of 37 °C.

Figure 22a shows the evolution of the open circuit potential (OCP) for the as-received titanium and the as-received titanium coated with zein. OCP for the uncoated titanium slightly decreased at the beginning of the measurement and after about 5000 s was stable in time and equaled −0.03 V. Stationary corrosion potential slightly increased for the coated titanium from −0.30 V and reached a stable value after about 20,000 s equaling −0.16 V. The results of E_ocp_ show that the coated alloy exhibited a more active potential (lower E_ocp_ = −0.30 V) than the uncoated titanium substrate, indicating its higher susceptibility to corrosion. The anodic cyclic potentiodynamic polarization curves of both samples in Ringer’s solution are presented in Figure 22b. A passive behavior with a large passive potential range was exhibited by both pure titanium and coated titanium. The current density in the anodic domain was slightly higher on the coated sample than on the pure titanium. The passive current density (i_p_) was reduced from 2.43 µA/cm^2^ for the coated sample to 0.50 µA/cm^2^ for the uncoated one, while the cathodic—anodic transition increased from −0.39 V to −0.12 V, respectively. Figure 22c presents the impedance spectra (Nyquist plot) obtained for the as-received titanium at an open circuit potential. The Nyquist diagram exhibits depressed and incomplete semicircles. The Ti alloys/aqueous solution interface was described previously by several equivalent circuit models, depending on the structure of the oxide layer [54,55,56]. In accordance with the XPS investigations of the as-received titanium performed in this work, the existence of a compact oxide layer was represented by an equivalent circuit with one time constant [54,55,56]. The EIS studies were carried out in order to describe the electrochemical interface of the titanium substrates in terms of an equivalent circuit. In order to adjust to a double-layer model for the surface coating, the equivalent circuit consisted of the constant phase element (CPE), the electrolyte resistance (R1) and the coating resistance (R2) (the equivalent Randles circuit). These results depict a predominantly capacitive behavior of the metal/solution interface, characteristic for passive materials with very high corrosion resistance.

The zein coatings had slightly worse corrosion resistance parameters compared to the uncoated metal. This result can be strongly connected with the presence of open pores with relatively high diameter up to 25 μm (Figure 17), which lead to easy penetration of the corrosive environment into the coating and cause damage. Xu et al. [57] showed that the rise of porosity inevitably deteriorates the corrosion resistance, and accordingly, the corrosion rate of the porous Ti-10Mo alloy increases exponentially. In order to improve coating quality, particularly to avoid the formation of open porosity in the coating resulting from water electrolysis, and thus to enhance the electrochemical corrosion resistance of the titanium coated with zein, further research will be focused on using pulsed direct current (PDC) and alternating current (AC) electrophoretic deposition.

## 4. Conclusions

(1) The EPD conditions for the deposition of pure zein coatings on the titanium substrates were established. To obtain homogeneous coatings, the ratio of water to ethanol in the zein solution should be approx. 10 vol % of water and 90 vol % of anhydrous ethanol. Higher water content resulted in nonuniform and cracked coatings, while higher ethanol content caused the precipitation of zein in the solution. The zein content in the solution should not be less than about 150 g/L and not higher than 200 g/L. Lower zein content led to inhomogeneous coatings, while higher content significantly increased the solution density;
(2) The adhesion of zein coatings to the titanium substrates strongly depended on their surface preparation. It was found that surface chemistry and surface features have a greater influence on the coating adhesion than the surface roughness of the substrates. The coating exhibited very good adhesion to the as-received and anodic oxidized substrates due to the presence of a 10 nm thick TiO_2_ layer on the surface and the presence of specific surface features, such as deep scratches and open porosity, respectively. In contrast, the adhesion of zein coatings to substrates chemically treated with HF and HNO_3_ acids or with H_2_O_2_ and H_2_SO_4_ acid or HCl and H_2_SO_4_ acids was poor even though the surface roughness parameters, R_a_ and R_q_, of these substrates were higher than that of the as-received titanium. In this case, the oxide layer present on the sample surface was thinner and consisted of different titanium oxides. The obtained results indicate that both adhesion mechanisms, mechanical interlocking and chemical bonding, are responsible for high coating adhesion to the underlying titanium substrates;
(3) The zein coatings were dense and exhibited higher surface topography development than the as-received substrate material. Sporadically, open pores on the surface and closed pores in the middle part of the coatings were observed;
(4) The zein coatings exhibited a hydrophilic nature and relatively high surface free energy (of 59.5 ± 3.8 mN/m), about 40% higher than that of the as-received substrate.
(5) The zein coated titanium exhibited slightly lower resistance to corrosion in Ringer’s solution at the temperature of 37 °C compared to the as-received titanium;

This work contributes knowledge in the EPD of zein on Ti substrates and on the effect of substrate preparation conditions on coating adhesion. It is expected that the result of the work provides an insight into the development of zein-based composite coatings by EPD.

## Figures and Tables

**Table 1:** Chemical compositions and pH of solutions used for electrophoretic deposition (EPD).

No	Zein (g/L)	Ethanol (vol %)	Distilled Water (vol %)	Glycerol (wt %)	pH
1	100	90	10	20	5.88
2	150	90	10	20	5.68
3	150	90	10	20	5.79
4	150	85	15	20	5.74
5	150	75	25	20	5.59
6	200	90	10	20	5.83

**Table 2:** Route of solutions preparation and EPD parameters.

No	Stirring Time (min)	Dispersion Time (min)	Stirring Velocity (rpm)	Voltage (V)	Deposition Time (min)
1	30	30	500	3, 5, 7, 10	5
2	30	30	500	3, 5, 7, 10	5
3	90	30	500	3, 5, 7, 10, 15, 20, 25, 30	5
4	90	30	500	3, 5, 7, 10, 15, 20, 25, 30	5
5	180	30	500	3, 5, 7, 10	5
6	90	30	1000	3, 5, 7	5

**Table 3:** Zeta potential of zein–ethanol–water solution at varying ethanol concentrations. The solid content in all solutions (pH 5.5–6.0) was diluted to 0.1 g/L.

Ethanol Content [vol %]	Zeta Potential [mV]
75	0.03 ± 0.21
85	2.22 ± 0.57
90	6.09 ± 0.84

**Table 4:** The adhesion class of zein coatings to the substrate after various surface preparations.

Ti Surface Preparation	Coatings		Adhesion Class, According to ASTM D3359-17
	Content of Zein in Solution (g/L)	Deposition Time (min)	
As-received	150	5	0B
As-received	200	5	4B
Treatment A	200	5	0B
Treatment B	200	2.5	4B
Treatment B	200	5	4B
Treatment C	200	2.5	2B
Treatment C	200	5	2B
Treatment D	200	2.5	0B
Treatment D	200	5	0B

**Table 5:** Parameters of surface topography for the as-received substrate and substrates chemically treated according to procedures A–D.

Parameter	As-Received	A	B	C	D
R_a_ [μm]	0.42 ± 0.10	0.46 ± 0.02	0.53 ± 0.04	0.51 ± 0.04	0.49 ± 0.04
R_q_ [μm]	0.57 ± 0.10	0.59 ± 0.02	0.76 ± 0.08	0.67 ± 0.06	0.67 ± 0.08
R_max_ [μm]	22.8 ± 5.7	5.6 ± 0.9	10.8 ± 1.7	9.1 ± 1.6	8.0 ± 0.8

## Data Availability

The data presented in this study are available on request from corresponding author.
